# Lebensbedrohliche euglykämische Ketoacidose unter Therapie mit Empagliflozin beim kardiochirurgischen Patienten: seltener Einzelfall oder zukünftig ein häufiges Problem?

**DOI:** 10.1007/s00101-024-01406-4

**Published:** 2024-04-18

**Authors:** H. Wachter, C. von Loeffelholz, D. O. Thomas-Rüddel, S. Bargenda, A. L. Birkenfeld, M. Bauer, J. Ehler

**Affiliations:** 1 Klinik für Anästhesiologie und Intensivmedizin, Universitätsklinikum Jena, Am Klinikum 1, 07749 Jena, Deutschland; 2 Klinik für Herz- und Thoraxchirurgie, Universitätsklinikum Jena, Jena, Deutschland; 3 Klinik für Diabetologie, Endokrinologie, Nephrologie, Innere Medizin IV, Universitätsklinikum Tübingen, Tübingen, Deutschland; 4 Deutsches Zentrum für Diabetesforschung (DZD e. V.), Neuherberg, Deutschland

## Anamnese

Ein 59-jähriger männlicher Patient stellte sich mit pektanginösen Beschwerden vor. Per Herzkatheteruntersuchung wurde bei schwerer koronarer Dreigefäßerkrankung die Indikation zur kardiochirurgischen Versorgung gestellt. Die linksventrikuläre Ejektionsfraktion (LVEF) betrug 52 %, ohne regionale Wandbewegungsstörungen. Vorerkrankungen umfassten einen Diabetes mellitus Typ 2, arterielle Hypertonie und Dyslipidämie. Die Vormedikation bestand aus Aspirin, Atorvastatin, Bisoprolol, Empagliflozin, Irbesartan und Metformin. Die Versorgung erfolgte in Off-Pump-Coronary-Artery-Bypass(OPCAB)-Technik, drei koronare Bypässe wurden angelegt und der Patient im Fast-Track-Verfahren auf die Intermediate-Care-Station verlegt.

## Untersuchungsbefund

Am ersten postoperativen Tag (POD) entwickelte er eine Schocksymptomatik mit Sinustachykardie (120–150/min), Tachypnoe mit vertiefter (Kußmaul‑)Atmung (22–30/min), Kaltschweißigkeit, arterieller Hypotension mit Katecholaminpflichtigkeit (max. 0,3 µg/kgKG und min Noradrenalin) sowie Somnolenz.

## Diagnostik

Das 12-Kanal-EKG und das hochsensitive Troponin I waren postoperativ ohne Hinweise auf eine Ischämie, jedoch war echokardiographisch eine hyperdynamische Kreislaufsituation mit Hypovolämie nachweisbar. Die Ventrikelfunktion zeigte sich ohne neue Wandbewegungsstörungen stabil; Rechtsherzbelastung und Perikardtamponade wurden ausgeschlossen. Sichere Hinweise für eine Infektion bzw. Sepsis ergaben sich aus den klinischen und apparativen Befunden (z. B. Röntgenuntersuchung des Thorax) nicht. Seit Operationsende auffällig war eine in den arteriellen Blutgasanalysen (BGA) nachweisbare metabolische Acidose (pH 7,24, Anionenlücke max. 15,0 mmol/l, Tab. 1). Diurese und Retentionsparameter waren normwertig; per Urinstreifentest waren Ketonkörper hochpositiv nachweisbar.

**Table 1:** 

Tag	OD				1. POD				
Uhrzeit	7:30	11:15	18:00	21:45	03:30	05:30	08:00	15:15	16:15
pH-Wert	7,40	7,23	7,24	7,23	7,25	7,24	7,24	7,28	7,31
SBC (mmol/l)	24,10	18,40	15,90	14,10	13,60	13,10	13,10	14,90	16,10
BE (mmol/l)	−0,20	−7,50	−10,90	−13,50	−14,20	−15,00	−15,00	−12,20	−10,5
pCO_2_ (kPa) (mm Hg)	5,28 39,60	6,34 47,55	4,91 36,83	4,25 31,88	3,67 27,53	3,57 26,78	3,57 26,78	3,80 28,50	3,87 29,03
K^+^ (mmol/l)	3,40	3,80	4,20	4,20	3,90	3,70	4,40	4,30	4,90
Glucose (mmol/l) (mg/dl)	7,50 135	9,30 168	7,80 141	8,50 153	10,40 187	10,00 180	9,70 175	11,60 209	10,7 193
Lactat (mmol/l)	0,40	0,60	1,20	1,10	1,20	1,20	1,00	1,10	1,00
AG (mmol/l)	10,90	12,60	12,10	14,90	14,4	9,90	15,00	9,10	7,90

## Therapie und Verlauf

Die echokardiographisch gesehene Hypovolämie wurde zunächst bedarfsgerecht mittels kristalloidem Volumenersatz behandelt. Bei anhaltender Tachykardie wurde eine Frequenzkontrolle mittels i.v. verabreichtem Metoprolol versucht, die allerdings nicht zur klinischen Besserung führte. Aufgrund der Klinik und Medikation lag der Verdacht einer empagliflozininduzierten euglykämischen Ketoacidose (Blutzucker 9,7 mmol/l; 175 mg/dl) nahe. Empagliflozin war 24 h präoperativ zuletzt eingenommen worden. Therapeutisch wurde kontinuierlich Insulin (0,1 IE/kgKG und h) unter parenteraler Glucosesubstitution i.v. appliziert, Kalium und Phosphat wurden subsituiert. Das Monitoring erfolgte via stündlicher BGA. Die Kreislaufparameter und das Vigilanzniveau normalisierten sich daraufhin zügig; die metabolische Störung war innerhalb von 20 h regredient (Tab. 1).

## Diskussion

**Figure 1:**
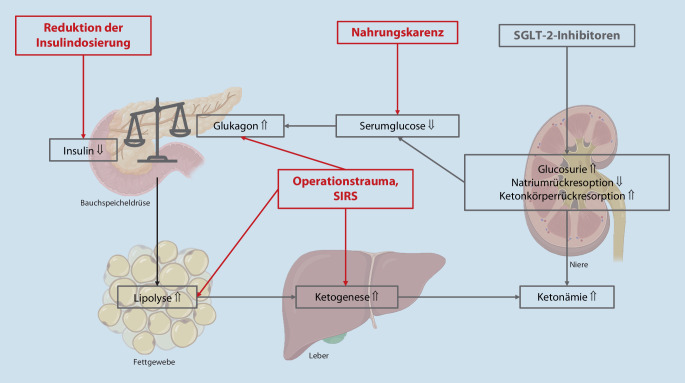

Sodiumglucosekotransporter-2-Inhibitoren (SGLT-2-Hemmer) sind zur Therapie des Diabetes mellitus Typ 2 zugelassen, aufgrund günstiger Beeinflussung klinischer Diabetesendpunkte auch primär [1, 2]. Seit 2021 ist Empagliflozin für die Herzinsuffizienztherapie ohne Diabetes verfügbar, seit 2023 auch bei leicht reduzierter Ejektionsfraktion (EF) [3, 4]. SGLT-2-Hemmer inhibieren den Natrium-2-Glucose-Kotransport im distalen Tubulus, was renale Glucoseverluste triggert und somit insulinunabhängig die Serumglucose senkt sowie zu osmotischer Diurese führt (Abb. 1). Die reduzierte Insulinausschüttung erhöht den Glukagonspiegel, mit der Folge gesteigerter Lipolyse und Ketogenese [5].

Triggerfaktoren, z. B. ein durch ein operationstraumainduziertes Systemic Inflammatory Response Syndrome (SIRS), können unter SGLT-2-Hemmern via veränderter Glukagon-Insulin-Ratio euglykämische Ketoacidosen induzieren, wovor die Food and Drug Administration bereits 2015 warnte [6–8]. Die perioperative Nahrungskarenz reduziert die Insulin- und erhöht die Glukagonspiegel, was sich im Falle reduzierter Insulingaben potenziert, zugleich steigt die renale Ketonkörperrückresorption (Abb. 1). Die exakte Pathophysiologie der euglykämischen Ketoacidose ist bisher nicht geklärt [9].

Die Diagnosestellung erfolgt meist verspätet. Die Serumglucose liegt häufig bei < 250 mg/dl (13,9 mmol/l), im vorliegenden Fall sogar bei < 180 mg/dl (10 mmol/l) [10]. Urinketone sind aufgrund erhöhter renaler Rückresorption (Abb. 1) nicht regelhaft erhöht [10]. Der Nachweis von Serum-β-Hydroxybutyrat und metabolischer Acidose mit Anionenlücke repräsentiert eine diagnostische Alternative [11].

Haupttherapieziel ist der zügige Ausgleich der metabolischen Acidose, inklusive Anionenlücke < 12 mmol/l [11]. Die i.v.-Insulingabe dient der Balancierung der Insulin-Glukagon-Ratio; sie hemmt Lipolyse und Ketogenese [12]. Zur Hypoglykämievermeidung ist die parallele Glucosesubstitution indiziert, bei langjährigem Diabetes besteht die Gefahr einer relativen Hypoglykämie [13]. Engmaschiges Monitoring mit stündlichen BGA-Kontrollen ist obligat, Blutzuckerziele von 140–200 mg/dl (7,7–11,0 mmol/l) sind akzeptabel, bei Diabetikern auf Basis der aktuellen internationalen Datenlage auch < 215 mg/dl (< 11,9 mmol/l) sowie laut aktueller Vorgabe der DIVI sogar < 250 mg/dl (< 13,9 mmol/l) [12, 14].

Konsequentes Absetzen der SGLT-2-Inhibitoren 48–72 h vor elektiven Eingriffen ist empfohlen; die Wiederaufnahme der Therapie erfolgt nach kaloriendeckender oraler Nahrungsaufnahme [15].

Aufgrund erweiterter Zulassung für die Herzinsuffizienztherapie ist in der perioperativen Herzchirurgie eine Zunahme an Fällen zu erwarten [16]. Diabetische Ketoacidosen gehen mit erhöhter Mortalität einher, im Zusammenhang mit SGLT-2-Inhibitoren bisher nur vereinzelt [17]. Unter Intensivtherapiebedingungen mit Insulingaben und liberalen Blutglucosezielen ist die Prognose gut; wichtig ist die zeitnahe Diagnosestellung [11]. In der klinischen Routine ist das zeitnahe Pausieren der SGLT-2-Hemmer präoperativ häufig noch erschwert, da Patienten vor Elektiveingriffen teilweise erst kurz vor dem Operationstermin stationär einbestellt werden und das Zeitfenster von 48–72 h zum Absetzen bereits verstrichen ist. Als Konsequenz werden die klinikexternen Zuweiser kardiochirurgischer Patienten am Universitätsklinikum Jena nun systematisch hinsichtlich zeitgerechter Terminierung der SGLT-2-Therapie instruiert.

## Fazit für die Praxis

- SGLT-2-Inhibitoren sollten 48–72 h präoperativ abgesetzt werden („sick day rules“).
- Die antidiabetische Therapie erfolgt bedarfsweise mit Insulin.
- Bei indikationsgerechtem Einsatz der SGLT-2-Inhibitoren (Herzinsuffizienz, Diabetes mellitus) muss mit einer zunehmenden perioperativen Häufigkeit euglykämischer Ketoacidosen gerechnet werden.
